# Rivaroxaban plus antiplatelet therapy for coronary artery ectasia: 36-month outcomes and risk prediction from a retrospective cohort study

**DOI:** 10.3389/fcvm.2026.1815929

**Published:** 2026-07-15

**Authors:** Mengwei Feng, Yunjie Wu, Chaoqing Xie, Jingxian Xing, Deguang Wang, Xue Liang, Juan Liu, Hui Gao, Tao Geng

**Affiliations:** 1 Department of Cardiology, Cangzhou Central Hospital of Hebei Medical University, Cangzhou, Hebei, China; 2 The Second Hospital of Tianjin Medical University, Tianjin, China

**Keywords:** antiplatelet therapy, coronary artery ectasia (CAE), D-dimer, major adverse cardiovascular events (MACE), propensity score matching, risk prediction model

## Abstract

**Background:**

Coronary artery ectasia (CAE) is characterized by abnormal coronary dilation and slow flow, predisposing patients to thrombotic complications. Optimal long-term antithrombotic strategies for CAE remain undefined. This study aimed to evaluate the efficacy and safety of low-dose rivaroxaban combined with single antiplatelet therapy in patients with CAE.

**Methods:**

In this single-center retrospective cohort study, 312 patients with CAE were enrolled and followed for 36 months. Patients received either single antiplatelet therapy alone or in combination with low-dose rivaroxaban. Propensity score matching (1:1) was performed to balance baseline characteristics. The primary endpoint was major adverse cardiovascular events (MACE). Secondary analyses included changes in thrombotic, inflammatory, and myocardial injury biomarkers. Cox proportional hazards models, inverse probability weighting, competing risk models, and sensitivity analyses were conducted to assess robustness.

**Results:**

After propensity score matching (124 vs. 124), combination therapy was associated with a significantly lower risk of 36-month MACE compared with antiplatelet therapy alone (8.1% vs. 21.8%; HR = 0.34, 95% CI: 0.19–0.62; *P* < 0.001), corresponding to an absolute risk reduction of 13.7% and a number needed to treat of 7.3. The benefit was more pronounced in patients with diffuse ectasia (Markis I/II: HR = 0.21, 95% CI: 0.09–0.49; interaction *P* = 0.02) and elevated baseline D-dimer (≥0.8 mg/L: HR = 0.18, 95% CI: 0.08–0.41; interaction *P* = 0.01). Improvements in D-dimer, inflammatory markers, and myocardial injury biomarkers were greater in the combination group. Total bleeding rates were not significantly different between groups, and no fatal bleeding occurred. Net clinical benefit favored combination therapy. Results remained consistent across multiple sensitivity analyses. A simple risk prediction model based on D-dimer, Markis classification, and treatment regimen was developed in the same cohort (C-index 0.78) but has not been externally validated.

**Conclusions:**

In patients with CAE, low-dose rivaroxaban combined with single antiplatelet therapy was associated with lower long-term MACE risk without a significant increase in major bleeding. The benefit appeared particularly pronounced in patients with diffuse ectasia and higher thrombotic burden. The simple predictive model based on D-dimer, Markis classification, and treatment regimen may aid in individualized antithrombotic decision-making, but requires external validation.

## 1 Introduction

Coronary artery ectasia (CAE) is a specific type of coronary artery lesion characterized by localized or diffuse dilation of the coronary artery lumen exceeding 1.5 times the diameter of the adjacent normal segment (1). With the widespread use of coronary angiography and coronary computed tomography angiography, the detection rate of CAE has gradually increased. Previous studies have reported a detection rate of approximately 1%–5% among patients undergoing coronary angiography, with a male predominance and frequent association with varying degrees of coronary atherosclerotic lesions (2). Unlike simple coronary stenosis, the pathophysiological features of CAE primarily involve abnormal vascular dilation, hemodynamic changes, and slowed blood flow. Blood stasis and vortex formation predispose to local thrombosis and distal embolization, thereby increasing the risk of adverse cardiovascular events such as acute coronary syndromes (3). Multiple clinical observations indicate that patients with CAE can experience recurrent myocardial ischemia symptoms even in the absence of severe stenotic lesions, leading to poor long-term prognosis (4–6). Therefore, optimizing antithrombotic strategies to reduce thrombotic risk is a key issue in CAE management.

Currently, there is no unified treatment guideline specifically for CAE. Clinical practice often follows the treatment principles for coronary atherosclerotic heart disease, primarily based on antiplatelet therapy. However, given the significant slow coronary flow and hypercoagulable tendency in patients with CAE, whether single antiplatelet therapy is sufficient to adequately inhibit thrombosis remains controversial. Some studies suggest that appropriate intensification of anticoagulation on top of antiplatelet therapy might help reduce thrombotic burden and ischemic events (7, 8), but the existing evidence is limited, particularly lacking systematic research in the CAE population. Rivaroxaban is a direct factor Xa inhibitor that exerts its anticoagulant effect by blocking the coagulation cascade. In recent years, multiple studies have confirmed that low-dose rivaroxaban combined with antiplatelet therapy can improve cardiovascular outcomes in patients with stable coronary artery disease and peripheral artery disease, with an acceptable safety profile (9). A 2024 updated systematic review by Amirpour et al. (10) summarized evidence from 25 studies and concluded that antiplatelet therapy (single or dual) combined with anticoagulation may provide more efficient protection against MACE in CAE patients, although the authors acknowledged that the evidence base was largely derived from case reports and case series, with only one randomized trial available. This underscores the urgent need for higher-quality evidence, particularly long-term outcome data, to guide antithrombotic decision-making in this population.

These COMPASS trials demonstrated that in patients with stable atherosclerosis, rivaroxaban 2.5 mg twice daily plus aspirin significantly reduced the risk of major adverse cardiovascular events compared to aspirin alone (9, 11). However, it is important to acknowledge that both trials specifically excluded patients with isolated coronary artery ectasia or were conducted in populations with documented atherosclerotic disease; therefore, direct extrapolation of their findings to the CAE population lacks supporting evidence and represents a key knowledge gap.

Given the unique pathophysiological mechanisms and hemodynamic characteristics of CAE, previous studies have largely included such patients within the broader coronary artery disease population, lacking specific clinical data. A recent exploratory randomized controlled trial in patients with acute coronary syndrome complicated by CAE showed that compared with dual antiplatelet therapy, clopidogrel plus rivaroxaban did not significantly reduce the primary cardiovascular endpoint but observed a reduction in myocardial infarction recurrence rate and shortened fibrin clot lysis time (12). While the aforementioned RCT provided valuable short-term (12-month) data, its limited sample size (*n* = 80) and short follow-up period precluded assessment of long-term efficacy and safety. Moreover, that study enrolled only patients with acute coronary syndrome (ACS) and did not specifically examine patients with stable CAE or those with varying morphological subtypes (Markis classification). The present retrospective cohort study addresses these gaps by: (1) extending follow-up to 36 months to evaluate durability of treatment benefit; (2) including both stable and post-ACS CAE patients, reflecting real-world clinical practice; (3) specifically analyzing effect modification by Markis classification (diffuse vs. localized ectasia) and baseline thrombotic burden (D-dimer), thereby identifying patient subgroups most likely to benefit; and (4) developing a simple risk prediction model to facilitate individualized decision-making. Thus, our study complements the existing RCT by providing longer-term observational evidence and hypothesis-generating insights for treatment stratification, which may inform the design of future adequately powered randomized trials in the CAE population.

Based on this background, this study retrospectively analyzed clinical data from patients with CAE in our hospital to compare the 36-month long-term efficacy and safety of single antiplatelet therapy vs. single antiplatelet therapy combined with rivaroxaban, aiming to provide evidence-based references for optimizing antithrombotic strategies in patients with CAE.

## 2 Materials and methods

### 2.1 Study design and participants

This was a single-center retrospective cohort study, consecutively enrolling patients who underwent coronary angiography and were diagnosed with CAE at Cangzhou Central Hospital between January 2021 and June 2022. Antithrombotic regimens were determined by the treating physicians at discharge based on individual patient conditions and bleeding risk. To document potential treatment selection bias, we retrospectively reviewed electronic medical records to record prespecified factors that could influence the decision to prescribe rivaroxaban, including: perceived high thrombotic burden (e.g., diffuse ectasia on angiogram, prior myocardial infarction with slow flow), perceived high bleeding risk (e.g., history of gastrointestinal bleeding, HAS-BLED score ≥3), physician specialty, and patient preference. These factors were not used as matching covariates but are reported to contextualize potential confounding.

It is important to emphasize that this approach—documenting but not adjusting for factors influencing treatment selection—does not quantitatively control for confounding by indication. The recorded factors (e.g., perceived thrombotic burden, bleeding risk, physician specialty, patient preference) represent subjective clinical judgments that are difficult to measure precisely and may have influenced treatment allocation in ways not captured by the propensity score model. Therefore, despite the comprehensive propensity score matching, residual confounding by indication cannot be excluded, and the results should be interpreted with this caveat prominently in mind. No instrumental variable analysis or quantitative bias analysis was performed to further adjust for unmeasured confounding.

Accordingly, patients were divided into a control group (single antiplatelet therapy) and a study group (single antiplatelet therapy combined with rivaroxaban). The study protocol was approved by the Medical Ethics Committee of Cangzhou Central Hospital (202425702). Due to the retrospective nature of the study and the anonymized data analysis, the requirement for informed consent was waived.

### 2.2 Diagnostic criteria

CAE was diagnosed based on coronary angiography results using internationally accepted standards (13): localized or diffuse dilation of a coronary artery with a lumen diameter ≥1.5 times the diameter of the adjacent normal segment. The extent of dilation was recorded according to the Markis classification (14): Type I: diffuse ectasia in two or three vessels; Type II: diffuse ectasia in one vessel and localized ectasia in another vessel; Type III: diffuse ectasia in only one vessel; Type IV: localized or segmental ectasia.

All angiograms were independently reviewed by two interventional cardiologists (blinded to clinical outcomes). Inter-observer agreement for Markis classification was assessed using Cohen's kappa coefficient (*κ* = 0.86, 95% CI: 0.79–0.93), indicating excellent agreement. Discordant cases (*n* = 6, 1.9%) were resolved by consensus with a third senior cardiologist.

### 2.3 Inclusion and exclusion criteria

Inclusion criteria were: (1) Age ≥18 years; (2) Diagnosis of CAE confirmed by coronary angiography; (3) Initiation of antithrombotic therapy post-discharge intended for at least 36 months; (4) Complete clinical and laboratory data; (5) Follow-up duration ≥36 months. Exclusion criteria were: (1) Definite indication for long-term anticoagulation (e.g., atrial fibrillation, mechanical heart valve replacement, venous thromboembolism), identified via systematic chart review using a combination of discharge diagnosis codes (ICD-10: I48 for atrial fibrillation, Z95.2 for mechanical heart valve, I26/I80 for venous thromboembolism) and medication reconciliation (warfarin or direct oral anticoagulants at therapeutic doses); (2) History of major bleeding event within the preceding 6 months; (3) Severe hepatic or renal insufficiency (estimated glomerular filtration rate <30 mL/min/1.73 m^2^); (4) Active malignancy, systemic inflammatory disease, or acute infection; (5) Pregnancy or breastfeeding; (6) Incomplete follow-up data or loss to follow-up.

**Figure 1:**
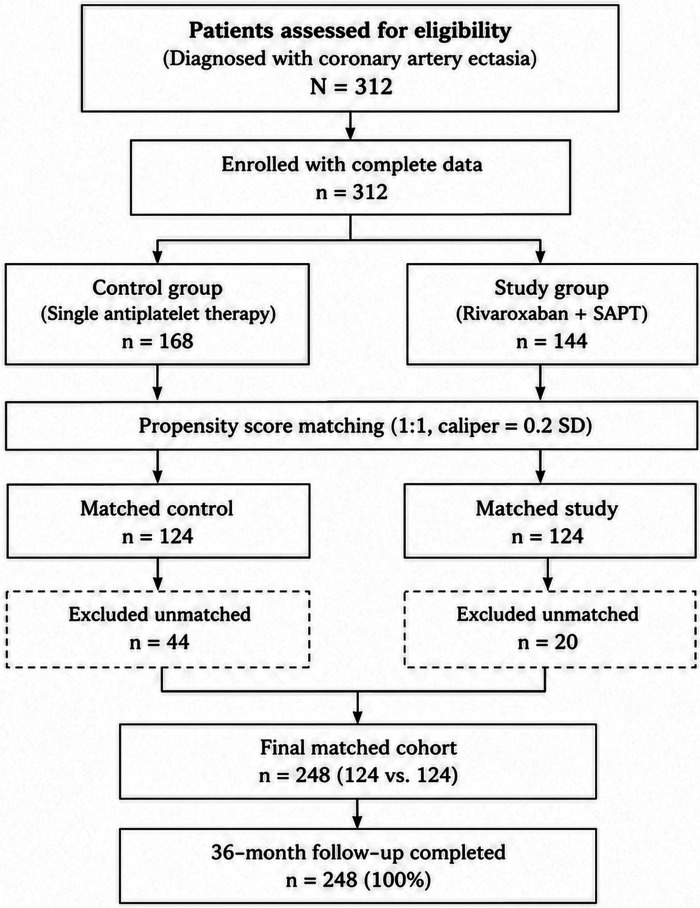
Study flowchart. Patients were enrolled between January 2021 and June 2022 and followed for 36 months.

All patients with any missing data (including incomplete laboratory results, missing medication adherence records, or loss to follow-up) were excluded *a priori* to achieve complete case analysis. This approach was pre-specified to avoid missing data imputation assumptions; the flow diagram (Figure 1) details the number of exclusions. Consequently, the final matched cohort reported zero missing data by design.

### 2.4 Treatment regimens

All patients received standardized comprehensive treatment according to guidelines for secondary prevention of coronary atherosclerotic heart disease (15), including statins (atorvastatin 20–40 mg/d or rosuvastatin 10–20 mg/d), beta-blockers (metoprolol or bisoprolol), angiotensin-converting enzyme inhibitors/angiotensin II receptor blockers (based on blood pressure and cardiac function), and lifestyle interventions (smoking cessation, low-salt low-fat diet, weight control, regular exercise). The control group received single antiplatelet therapy after discharge, including aspirin 100 mg/d (Bayer Healthcare, China Approval No. J20130078) or clopidogrel 75 mg/d (Sanofi, China Approval No. J20180029, for patients with aspirin intolerance or gastrointestinal reactions), orally once daily. The choice of antiplatelet agent was individualized by the attending physician based on the patient's medication history, tolerance, and bleeding risk. The study group received rivaroxaban (Bayer Healthcare, China Approval No. J20180076) in addition to single antiplatelet therapy (aspirin or clopidogrel, same regimen as control group). The standard dose was 2.5 mg twice daily. For some patients with low bleeding risk (HAS-BLED score <3) and body weight >80 kg, a dose of 10 mg once daily could be used. Dose selection was based on a comprehensive assessment of renal function, age, weight, and bleeding risk: The specific eGFR categories (≥50, 30–49 mL/min/1.73 m^2^) were recorded for all patients to enable subgroup analyses of efficacy and safety according to baseline renal function. For patients with eGFR 30–49 mL/min/1.73 m^2^ (*n* = 27, 10.9% of matched cohort), no empirical dose reduction was mandated based on current guidelines (which recommend caution but do not contraindicate rivaroxaban 2.5 mg bid), but renal function was monitored every 3 months. The proportion of patients with moderate renal impairment was similar between groups. Subgroup analyses by eGFR category were performed to assess consistency of efficacy and safety. Rivaroxaban was initiated at discharge, with a recommendation to continue for at least 36 months. Routine coagulation monitoring was not performed during treatment, but renal function and bleeding risk were assessed regularly during follow-up.

The choice of antiplatelet agent (aspirin vs. clopidogrel) was recorded, and exploratory subgroup analyses were planned to assess whether the treatment effect of adding rivaroxaban differed according to the specific antiplatelet agent. We acknowledge that pooling aspirin and clopidogrel in the single antiplatelet background could introduce heterogeneity. However, as prespecified, we performed exploratory subgroup analyses. Given the small number of patients receiving clopidogrel (*n* = 30 in the study group, *n* = 28 in the control group), the study was not powered to detect differential treatment effects; descriptive results are presented in Supplementary Table S1, showing directionally consistent effects.

### 2.5 Data extraction and quality control

Data were extracted from the hospital's electronic medical record system using a standardized electronic case report form. Before extraction, two researchers (both attending physicians in cardiology) underwent unified training to ensure a consistent understanding of the definitions and extraction criteria for each variable. Data extraction covered the following domains: demographic characteristics (age, sex, body mass index), comorbidities (hypertension, diabetes mellitus, hyperlipidemia, smoking history), prior cardiovascular history (myocardial infarction, percutaneous coronary intervention, stroke/transient ischemic attack), Markis classification (I/II/III/IV), discharge medications (antiplatelet agents, statins, beta-blockers, ACEI/ARB), laboratory results (thrombotic/coagulation markers, inflammatory markers, myocardial injury markers at baseline and 12 months), and adverse events during follow-up (dates and specific types of MACE, dates, sites, and BARC types of bleeding events). Extraction was performed independently by two researchers, followed by cross-checking. Inter-rater reliability was assessed on a random 10% sample (*n* = 31 patients) using Cohen's kappa for categorical variables and intraclass correlation coefficient for continuous variables; agreement was excellent (*κ* ≥ 0.85 for all categorical variables, ICC ≥ 0.92 for continuous variables). In case of discrepancies, the two researchers jointly reviewed the original medical records for confirmation, and a third senior physician made the final decision if necessary. Missing data were initially recorded, then traced through telephone or outpatient follow-up by designated personnel; if unobtainable, the case was excluded (no missing data in this study).

### 2.6 Propensity score matching

To reduce confounding bias, propensity score matching was used to balance baseline differences between groups. A multivariable logistic regression model was constructed to estimate propensity scores, with the receipt of rivaroxaban combination therapy as the dependent variable, including the following covariates: age (continuous), sex, BMI (continuous), hypertension, diabetes, hyperlipidemia, current smoking, prior myocardial infarction, prior PCI, prior stroke/TIA, Markis classification (Type I/II vs. III/IV), statin use at discharge, beta-blocker use, ACEI/ARB use, and HAS-BLED score (continuous). The inclusion of HAS-BLED score (components: hypertension, abnormal renal/liver function, stroke, bleeding history or predisposition, labile INR, elderly >65 years, drugs/alcohol concomitantly) addresses the concern that “bleeding risk” influenced treatment allocation. Covariate balance was assessed using absolute standardized mean differences (SMD), with SMD <0.1 considered well-balanced. For variables not included in the model (e.g., baseline D-dimer, hs-CRP), balance was verified post-matching by calculating SMD; SMD values were <0.1 for all variables except PT (0.11), indicating generally good balance. SMD values are reported in Table 1. Nearest neighbor matching was performed in a 1:1 ratio with a caliper width of 0.2 times the standard deviation of the logit of the propensity score. Matching effectiveness was assessed using standardized mean differences (<0.1 considered good balance) and density plots of propensity scores. Matching was performed using the MatchIt package in R 4.2.2.

**Table 1:** Baseline characteristics before and after propensity score matching.

Characteristic	Before matching			After matching			
	Control (*n* = 168)	Study (*n* = 144)	*P*-value	Control (*n* = 124)	Study (*n* = 124)	*P*-value	SMD
Demographics							
Age (years), mean ± SD	61.2 ± 9.4	62.5 ± 8.8	0.203	61.5 ± 9.2	62.1 ± 8.7	0.599	0.07
Male, *n* (%)	112 (66.7)	95 (66.0)	0.894	84 (67.7)	85 (68.5)	0.89	0.02
BMI (kg/m^2^), mean ± SD	24.3 ± 3.3	24.8 ± 3.1	0.165	24.5 ± 3.2	24.8 ± 3.1	0.455	0.1
Comorbidities, *n* (%)							
Hypertension	93 (55.4)	98 (68.1)	0.021	72 (58.1)	75 (60.5)	0.701	0.05
Diabetes mellitus	42 (25.0)	45 (31.3)	0.212	32 (25.8)	35 (28.2)	0.667	0.05
Hyperlipidemia	74 (44.0)	78 (54.2)	0.072	58 (46.8)	62 (50.0)	0.611	0.06
Current smoking	53 (31.5)	51 (35.4)	0.466	41 (33.1)	43 (34.7)	0.788	0.03
Previous MI	29 (17.3)	32 (22.2)	0.266	23 (18.5)	26 (21.0)	0.63	0.06
Previous PCI	38 (22.6)	41 (28.5)	0.227	31 (25.0)	33 (26.6)	0.772	0.04
Previous stroke/TIA	14 (8.3)	16 (11.1)	0.4	11 (8.9)	13 (10.5)	0.67	0.05
Markis classification, *n* (%)			0.047			0.938	
Type I	24 (14.3)	27 (18.8)		19 (15.3)	21 (16.9)		0.04
Type II	40 (23.8)	44 (30.6)		32 (25.8)	31 (25.0)		0.02
Type III	58 (34.5)	41 (28.5)		42 (33.9)	39 (31.5)		0.05
Type IV	46 (27.4)	32 (22.2)		31 (25.0)	33 (26.6)		0.04
Medications at discharge, *n* (%)							
Aspirin	129 (76.8)	110 (76.4)	0.934	96 (77.4)	94 (75.8)	0.765	0.04
Clopidogrel	39 (23.2)	34 (23.6)	0.934	28 (22.6)	30 (24.2)	0.765	0.04
Statin	159 (94.6)	138 (95.8)	0.618	118 (95.2)	120 (96.8)	0.517	0.08
*β*-blocker	117 (69.6)	106 (73.6)	0.436	89 (71.8)	92 (74.2)	0.667	0.05
ACEI/ARB	99 (58.9)	96 (66.7)	0.156	76 (61.3)	80 (64.5)	0.6	0.07
Baseline laboratory, mean ± SD							
D-Dimer (mg/L)	0.82 ± 0.23	0.85 ± 0.24	0.255	0.83 ± 0.22	0.84 ± 0.23	0.728	0.04
Fibrinogen (g/L)	4.22 ± 0.68	4.29 ± 0.72	0.371	4.24 ± 0.66	4.26 ± 0.69	0.816	0.03
PT (s)	12.1 ± 1.0	12.3 ± 0.9	0.064	12.2 ± 0.9	12.3 ± 0.9	0.385	0.11
APTT (s)	29.6 ± 3.5	30.0 ± 3.6	0.315	29.8 ± 3.3	29.9 ± 3.4	0.814	0.03
IL-6 (pg/mL)	11.7 ± 3.9	12.3 ± 3.8	0.166	11.9 ± 3.8	12.1 ± 3.7	0.676	0.05
TNF-α (pg/mL)	18.3 ± 5.0	19.1 ± 5.2	0.162	18.5 ± 4.8	18.8 ± 5.0	0.636	0.06
hs-CRP (mg/L)	6.4 ± 2.3	6.9 ± 2.5	0.064	6.5 ± 2.2	6.7 ± 2.4	0.498	0.09
cTnI (ng/mL)	0.14 ± 0.06	0.15 ± 0.06	0.14	0.15 ± 0.06	0.15 ± 0.06	1	0
NT-proBNP (pg/mL)	610.2 ± 192.5	635.8 ± 198.3	0.241	618.5 ± 188.3	623.7 ± 191.5	0.83	0.03

BMI, body mass index; MI, myocardial infarction; PCI, percutaneous coronary intervention; TIA, transient ischemic attack; ACEI, angiotensin-converting enzyme inhibitor; ARB, angiotensin receptor blocker; PT, prothrombin time; APTT, activated partial thromboplastin time; IL-6, interleukin-6; TNF-α, tumor necrosis factor-α; hs-CRP, high-sensitivity C-reactive protein; cTnI, cardiac troponin I; NT-proBNP, N-terminal pro-B-type natriuretic peptide.

SMD, standardized mean difference; SMD <0.1 indicates good balance.

The selection of covariates for propensity score matching followed established guidelines for avoiding overadjustment bias. We intentionally excluded baseline D-dimer, coagulation parameters (fibrinogen, PT, APTT), and inflammatory markers (IL-6, TNF-α, hs-CRP) from the propensity score model for the following reasons: (1) these laboratory markers were measured at discharge after the antithrombotic regimen had been initiated in most patients, and thus could have been influenced by the index hospitalization treatments, violating the temporal precedence requirement for propensity score covariates; (2) these markers lie on the causal pathway between disease severity and treatment selection—including them would risk balancing post-treatment variables and attenuating the estimated treatment effect; (3) leaving them unmatched allowed us to subsequently evaluate their role as effect modifiers and to assess treatment-induced changes in these biomarkers as secondary endpoints.

### 2.7 Laboratory measurements

Fasting venous blood samples (10 mL) were collected for baseline laboratory measurements at the time of hospital admission prior to any antithrombotic therapy (i.e., before diagnostic coronary angiography and before initiation of in-hospital antiplatelet or anticoagulant treatment). Follow-up samples were collected after 12 months of treatment during scheduled outpatient visits. Samples for thrombotic and coagulation markers were collected using tubes containing sodium citrate (1:9). Serum for inflammatory and myocardial markers was collected using tubes without anticoagulant. Specimens were allowed to stand at room temperature for 30 min, then centrifuged at 3,000 r/min for 10 min to separate plasma or serum, aliquoted into 1.5 mL Eppendorf tubes, and stored at −80 °C until analysis. All measurements were performed by the clinical laboratory of our hospital, strictly following reagent instructions, and were operated by a dedicated team of three trained laboratory technicians throughout the study period; monthly quality control checks and inter-technician correlation assessments (Pearson *r* > 0.95 for all analytes) ensured consistency. Thrombotic and coagulation markers were measured using an automated coagulation analyzer (CS-5100, Sysmex, Japan) with reagents. D-dimer was measured by immunoturbidimetry (reference range <0.5 mg/L), fibrinogen by the Clauss method (reference range 2.0–4.0 g/L), prothrombin time by the PT method (reference range 11–14 s), and activated partial thromboplastin time by the clotting method (reference range 25–35 s). Inflammatory markers, including interleukin-6 (sensitivity 0.1 pg/mL), tumor necrosis factor-α (sensitivity 0.5 pg/mL), and high-sensitivity C-reactive protein (sensitivity 0.01 mg/L), were measured using enzyme-linked immunosorbent assay kits (R&D Systems, USA). All samples were measured in duplicate within the same batch, and the mean values were used for analysis to minimize inter-batch variation. Myocardial injury markers, cardiac troponin I (chemiluminescence method, reference range <0.04 ng/mL) and N-terminal pro-B-type natriuretic peptide (chemiluminescence method, reference range <125 pg/mL), were measured using an automated chemiluminescence immunoassay analyzer (ADVIA Centaur, Siemens, Germany).

### 2.8 Follow-up and outcome definitions

All patients were followed up for 36 months from the discharge date (i.e., from January 2021–June 2022 to January 2024–June 2025). Follow-up methods included outpatient visits (at 1, 3, 6, 12, 18, 24, 30, 36 months) and telephone interviews. Medication adherence was assessed through pill counts and patient self-reporting; good adherence was defined as taking medication for ≥80% of the total follow-up days. The primary efficacy endpoint was major adverse cardiovascular events, defined as a composite of cardiac death, non-fatal myocardial infarction, non-fatal ischemic stroke, rehospitalization for myocardial ischemia, and repeat revascularization. Events were adjudicated independently by two cardiologists, recording the date and specific type of event, and cumulative event rates at 12, 24, and 36 months were calculated. Secondary efficacy endpoints were changes from baseline in thrombotic/coagulation markers, inflammatory markers, and myocardial injury markers after 12 months of treatment (Δ). The safety endpoint was bleeding events, classified as types 1–5 according to the Bleeding Academic Research Consortium (BARC) criteria (16). Per BARC, type 3 bleeding was defined as follows: type 3a — hemoglobin drop of 3 to <5 g/dL or any transfusion with overt bleeding; type 3b — hemoglobin drop ≥5 g/dL or cardiac tamponade or bleeding requiring surgical intervention; type 3c — intracranial hemorrhage, intraocular bleeding compromising vision, or intramuscular with compartment syndrome. The date of each bleeding event was recorded, and the timing relative to treatment initiation (months) was calculated. Discontinuation of rivaroxaban or antiplatelet therapy due to bleeding was defined as permanent or temporary cessation for >7 days directly attributable to a bleeding event. The reason, duration, and subsequent restart of therapy were documented. All bleeding events were adjudicated by two independent cardiologists. Net clinical benefit was defined as the composite of MACE and BARC type 3 major bleeding.

Pre-planned landmark analyses (at 12 and 24 months) were performed to assess the timing and persistence of treatment effect; results are presented in Supplementary Table S2.

### 2.9 Sample size

As this was a retrospective exploratory study, a formal prospective sample size calculation was not performed. However, based on previous research (17) and the higher thrombotic burden in CAE patients, we anticipated that the combination therapy group would have an approximately 40% reduction in MACE incidence compared to the control group.

To ensure that the final matched cohort would have adequate precision to detect a clinically meaningful difference, we performed a *post-hoc* power calculation based on the observed MACE rates (21.8% vs. 8.1%) in the matched cohort. With 124 patients per group, two-sided *α* = 0.05, and a 13.7% absolute risk reduction, the achieved power exceeded 99%. For a more conservative effect size (assuming a 30% relative risk reduction, corresponding to an absolute risk reduction from 21.8% to 15.3%), the study had 82% power. Thus, the final sample size was sufficient to address the primary research question. Extending follow-up to 36 months allowed further assessment of the persistence of long-term efficacy, and interim landmark analyses (12 and 24 months) were added to characterize the temporal evolution of treatment benefit.

The final matched cohort of 124 patients per group met this power requirement. Extending follow-up to 36 months allowed further assessment of the persistence of long-term efficacy.

### 2.10 Statistical analysis

Statistical analysis was performed using GraphPad Prism 9.0 and R 4.2.2. Continuous variables, all conforming to a normal distribution by Shapiro–Wilk test, were expressed as mean ± standard deviation. Categorical variables were expressed as frequencies and percentages. After propensity score matching, baseline comparisons between groups were performed using paired *t*-tests (continuous variables) or McNemar's test/conditional logistic regression (categorical variables). Within-group comparisons of laboratory markers before and after treatment were performed using paired *t*-tests, and between-group comparisons of changes (Δ) were performed using independent samples *t*-tests. Pearson correlation analysis was used to assess the correlation between ΔD-dimer and Δhs-CRP, and Fisher's z transformation was used to compare the correlation coefficients between groups. Time-to-event outcomes (MACE, net clinical benefit) were analyzed using Kaplan–Meier curves and compared using the log-rank test. Hazard ratios and 95% confidence intervals were calculated using Cox proportional hazards models (stratified by matched pairs), along with absolute risk reduction and number needed to treat. Prespecified subgroup analyses were performed for age (<65 vs. ≥65 years), sex, diabetes, hypertension, Markis classification (Type I/II vs. III/IV), and baseline D-dimer level (<0.8 mg/L vs. ≥0.8 mg/L). Within each subgroup, HRs and 95% CIs were calculated using Cox models, and heterogeneity was tested by including treatment-by-subgroup interaction terms. For safety analyses, bleeding event rates were compared between groups using McNemar's test. To verify the robustness of the primary findings, the following sensitivity analyses were performed: inverse probability of treatment weighting analysis, reanalysis after excluding patients receiving non-standard rivaroxaban doses, multivariable Cox regression in the unmatched cohort, Fine-Gray competing risk model, and per-protocol analysis. All tests were two-sided, and *P* < 0.05 was considered statistically significant.

To aid individualized clinical decision-making, a Cox proportional hazards regression model predicting 36-month MACE risk was developed in the propensity score-matched cohort (*n* = 248). Due to concerns about overfitting and instability with stepwise selection, we used a pre-specified variable selection approach based on clinical plausibility and univariable associations (*P* < 0.10). Candidate predictors included baseline D-dimer level (continuous, natural log-transformed), Markis classification (Type I/II vs. III/IV), treatment group (study vs. control), age, sex, BMI, comorbidities, smoking history, and prior cardiovascular history. Only three variables (log D-dimer, Markis classification, treatment group) met the pre-specified inclusion criterion and were entered into the final multivariable model. No automated stepwise algorithm was used.

Model discrimination was assessed using Harrell's C-index with 95% confidence intervals. Calibration was assessed using calibration plots and the Hosmer-Lemeshow test; optimism was corrected using bootstrapping (500 resamples). Decision curve analysis was performed to evaluate clinical utility. A nomogram was constructed for visual presentation.This model is exploratory and requires external validation in independent CAE cohorts before clinical application.

To assess the clinical utility of the model, decision curve analysis was performed to calculate the net benefit at different threshold probabilities. Net benefit was calculated as: NB = (True positives/n)—(False positives/n) × [Threshold probability/(1-Threshold probability)]. The net benefit of this model was compared against a simplified model based only on treatment group, a “treat-all” strategy, and a “treat-none” strategy over a threshold probability range of 0%–50%.

Based on the final model, a nomogram was constructed to visually display the predicted 36-month MACE probability for individual patients. The nomogram converted the regression coefficients of each variable into point scores ranging from 0 to 100. The points for each variable were summed to obtain a total score, which corresponded to a predicted probability. The predictive performance of the nomogram was validated using the C-index and calibration plot mentioned above.

To explore whether the correlation between ΔD-dimer and Δhs-CRP varied according to disease phenotype or thrombotic burden, stratified Pearson correlation analyses were performed according to Markis classification (Type I/II vs. III/IV) and baseline D-dimer level (<0.8 vs. ≥0.8 mg/L). Correlation coefficients were compared using Fisher's z-transformation.

## 3 Results

### 3.1 Study population and propensity score matching

A total of 312 patients diagnosed with CAE by coronary angiography between January 2021 and June 2022 were initially enrolled. According to post-discharge antithrombotic regimens, 168 patients received single antiplatelet therapy (control group) and 144 patients received rivaroxaban plus single antiplatelet therapy (study group). Prior to matching, patients in the study group had a higher prevalence of hypertension (68.1% vs. 55.4%, *P* = 0.021) and a greater proportion of diffuse ectasia (Markis type I/II: 52.8% vs. 41.7%, *P* = 0.047), indicating potential selection bias.

To minimize confounding effects, 1:1 propensity score matching was performed using a logistic regression model that included age, sex, body mass index, hypertension, diabetes mellitus, hyperlipidemia, smoking status, previous cardiovascular history, Markis classification, and baseline medication use. The matching algorithm employed a caliper width of 0.2 of the standard deviation of the logit of the propensity score. The matching procedure successfully created 124 well-balanced pairs (total 248 patients). After matching, all baseline characteristics were comparable between the two groups with no statistically significant differences (*P* > 0.05 for all variables), as shown in Table 1, and the study flowchart is shown in Figure 1.

Notably, despite not being included in the propensity score model, baseline D-dimer, coagulation, and inflammatory markers were well balanced between the two groups after matching (Table 1), suggesting that their exclusion did not compromise covariate balance.

**Figure 2:**
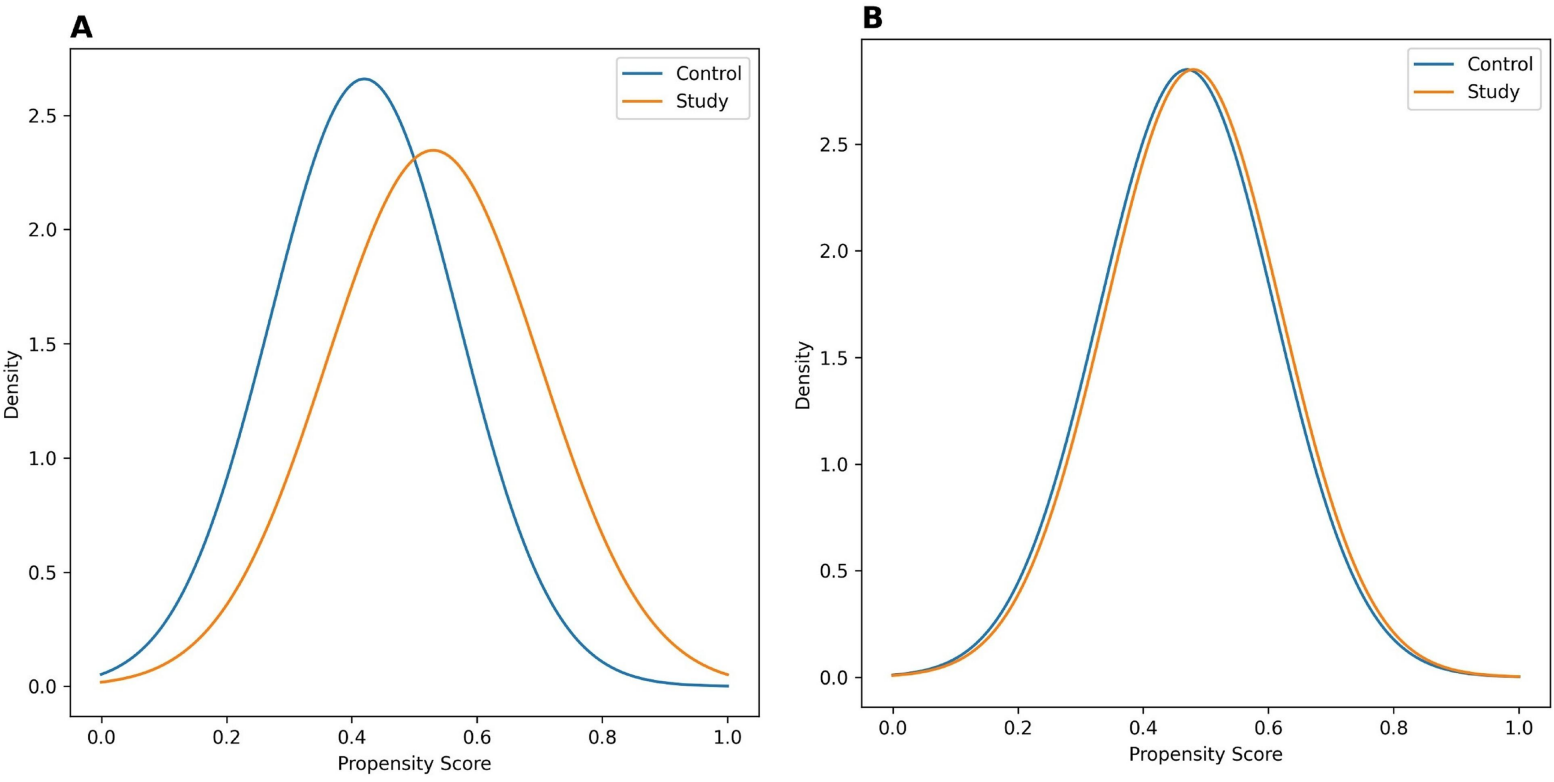
Distribution of propensity scores before and after matching. **(A)** Density plots of propensity scores before matching, showing a rightward shift in the study group. **(B)** Density plots after 1:1 matching, demonstrating near-perfect overlap between the two groups.

Figure 2 illustrates the distribution of propensity scores before and after matching. Prior to matching, the study group had a right-shifted distribution indicating higher propensity for receiving rivaroxaban (Figure 2A). After matching, the distributions were nearly identical, confirming successful balance (Figure 2B).

The matched cohort consisted of 248 patients (mean age 61.8 ± 8.9 years; 68.1% male). The distribution of Markis classification was similar between groups, with type III being the most frequent (control: 33.9%, study: 31.5%). Regarding antiplatelet selection, aspirin was used in 77.4% of control patients and 75.8% of study patients, while clopidogrel was used in the remaining patients due to aspirin intolerance or gastrointestinal concerns. Baseline laboratory parameters, including thrombotic markers, inflammatory markers, and myocardial injury markers, showed no significant intergroup differences, confirming successful matching (Table 1). Given the small number of patients receiving clopidogrel (*n* = 30 in the study group, *n* = 28 in the control group), the study was not powered to detect differential treatment effects between aspirin and clopidogrel.

In exploratory subgroup analyses according to background antiplatelet agent, the treatment effect of adding rivaroxaban appeared directionally consistent: aspirin subgroup HR = 0.36 (95% CI: 0.17–0.76), clopidogrel subgroup HR = 0.31 (95% CI: 0.08–1.25). However, the clopidogrel subgroup included only 30 patients in the study group and 28 in the control group; the wide confidence interval reflects limited statistical power. A formal interaction test was performed (*P* for interaction = 0.78), but this test is also underpowered and should be interpreted with caution. These results are descriptive and hypothesis-generating only.

In the matched cohort, the mean HAS-BLED score was 1.8 ± 0.7 in the control group and 1.9 ± 0.7 in the study group. The proportion of patients with HAS-BLED score <3 (low bleeding risk) was 95.2% (118/124) in the control group and 93.5% (116/124) in the study group. These data indicate that the study population was predominantly at low bleeding risk, consistent with the exclusion of patients with HAS-BLED ≥3 or a history of major bleeding.

#### 3.1.1 Renal function distribution

Among the 248 matched patients, 221 (89.1%) had eGFR ≥50 mL/min/1.73 m^2^, while 27 (10.9%) had eGFR between 30 and 49 mL/min/1.73 m^2^. No patient had eGFR <30 mL/min/1.73 m^2^ per exclusion criteria. The proportion of patients with eGFR 30–49 mL/min was similar between the study group (14/124, 11.3%) and the control group (13/124, 10.5%; *P* = 0.84). In the study group, among those with eGFR 30–49 mL/min, 12 patients (85.7%) received the standard dose of rivaroxaban 2.5 mg twice daily, and 2 patients (14.3%) received 10 mg once daily; For patients with eGFR 30–49 mL/min/1.73 m^2^ (*n* = 27, 10.9% of matched cohort), we followed the manufacturer's prescribing information and current guidelines, which recommend caution but do not mandate dose reduction for rivaroxaban 2.5 mg twice daily in this eGFR range. Nevertheless, to ensure safety, renal function was monitored every 3 months, and no patient experienced drug accumulation-related adverse events. No bleeding occurred in this subgroup that required dose adjustment or discontinuation.

#### 3.1.2 Rivaroxaban dosing distribution in the study group

Among the 124 patients in the study group (rivaroxaban plus single antiplatelet therapy), 113 patients (91.1%) received the standard low-dose regimen of rivaroxaban 2.5 mg twice daily, while 11 patients (8.9%) received the alternative regimen of 10 mg once daily. The alternative regimen was used only in patients with HAS-BLED score <3 and body weight >80 kg, as prespecified in the protocol. Baseline characteristics, including age, sex, body weight, eGFR, and Markis classification, were comparable between the two dosing subgroups (all *P* > 0.05), except for body weight which was higher in the 10 mg qd group by design (92.3 ± 6.7 kg vs. 75.6 ± 9.2 kg, *P* < 0.001).

Due to the small number of patients receiving 10 mg once daily (*n* = 11), formal comparative analysis of efficacy and safety between the two rivaroxaban regimens was not feasible. In descriptive comparisons, the 10 mg once daily subgroup (*n* = 11) had one MACE (9.1%) and two minor bleeding events (18.2%), while the 2.5 mg twice daily subgroup (*n* = 113) had nine MACE (8.0%) and 19 minor bleeding events (16.8%). Fisher's exact test yielded two-tailed *P* values of 0.99 for MACE and 0.68 for minor bleeding, but these comparisons are severely underpowered and should not be interpreted as evidence of equivalence. Given the small number of patients receiving 10 mg once daily (8.9% of the study group), no formal comparative analysis was feasible, and the two dose groups were pooled for the primary analysis after confirming no major differences in baseline characteristics.

Notably, the primary sensitivity analysis that excluded all 11 patients receiving the 10 mg qd regimen (along with 7 control patients with dose deviations) yielded a consistent treatment effect (HR = 0.33, 95% CI: 0.17–0.63; *P* < 0.001), indicating that the inclusion of alternative dosing did not bias the overall conclusion.

### 3.2 Improvements in thrombotic and coagulation parameters

After 12 months of treatment, both groups showed significant improvements in thrombotic and coagulation parameters compared to baseline. However, the study group demonstrated significantly greater improvements, as shown in Table 2.

**Table 2:** Comparison of thrombotic and coagulation parameters.

Parameter	Control group (*n* = 124)	Study group (*n* = 124)	Between-group	*P*-value
D-dimer (mg/L)				
Baseline	0.83 ± 0.22	0.84 ± 0.23		0.728
12 months	0.58 ± 0.17	0.39 ± 0.14		<0.001
Change (Δ)	−0.25 ± 0.15	−0.45 ± 0.18	*t* = 9.486	<0.001
Within-group *P*	<0.001	<0.001		
Fibrinogen (g/L)				
Baseline	4.24 ± 0.66	4.26 ± 0.69		0.816
12 months	3.62 ± 0.53	3.08 ± 0.47		<0.001
Change (Δ)	−0.62 ± 0.40	−1.18 ± 0.45	*t* = 10.378	<0.001
Within-group *P*	<0.001	<0.001		
PT (s)				
Baseline	12.2 ± 0.9	12.3 ± 0.9		0.385
12 months	13.1 ± 1.0	14.5 ± 1.2		<0.001
Change (Δ)	+0.9 ± 0.7	+2.2 ± 0.9	*t* = 12.724	<0.001
Within-group *P*	<0.001	<0.001		
APTT (s)				
Baseline	29.8 ± 3.3	29.9 ± 3.4		0.814
12 months	32.3 ± 3.6	36.2 ± 4.1		<0.001
Change (Δ)	+2.5 ± 2.1	+6.3 ± 2.8	*t* = 12.042	<0.001
Within-group *P*	<0.001	<0.001		

Data are presented as mean ± standard deviation. Δ indicates absolute change from baseline to 12 months.

All within-group *P* values remained <0.001 after Bonferroni correction (adjusted α = 0.00625).

D-dimer levels decreased substantially in both groups, but the reduction was markedly greater in the study group (Δ = −0.45 ± 0.18 mg/L vs. −0.25 ± 0.15 mg/L; *P* < 0.001). Similarly, fibrinogen levels showed a more pronounced decline in patients receiving rivaroxaban combination therapy (Δ = −1.18 ± 0.45 g/L vs. −0.62 ± 0.40 g/L; *P* < 0.001). Coagulation times were prolonged in both groups, reflecting the antithrombotic effect, with significantly greater prolongation of PT (Δ = +2.2 ± 0.9 s vs. +0.9 ± 0.7 s; *P* < 0.001) and APTT (Δ = + 6.3 ± 2.8 s vs. +2.5 ± 2.1 s; *P* < 0.001) in the study group.

These findings indicate that the addition of rivaroxaban to single antiplatelet therapy more effectively suppresses the hypercoagulable state characteristic of CAE patients, with the absolute changes suggesting a clinically meaningful intensification of antithrombotic effect.

The mean APTT in the study group at 12 months (36.2 ± 4.1 s) exceeded the upper reference limit of 35 s, indicating a measurable anticoagulant effect of rivaroxaban. However, this prolongation was modest and not associated with increased major bleeding (only one BARC type 3 bleeding occurred in the study group). No patient required dose reduction or discontinuation due to excessive coagulation parameter prolongation.

### 3.3 Synergistic reduction in inflammatory markers and thrombo-inflammatory correlation

Both treatment regimens significantly reduced inflammatory marker levels after 12 months. However, the study group exhibited markedly greater reductions in all measured inflammatory parameters (Table 3). The decrease in IL-6 was nearly twice as large in the study group compared to controls (Δ = −6.1 ± 2.5 pg/mL vs. −3.4 ± 2.1 pg/mL; *P* < 0.001). Similarly, TNF-α reduction was significantly greater with combination therapy (Δ = −8.1 ± 3.2 pg/mL vs. −4.3 ± 2.8 pg/mL; *P* < 0.001), as was hs-CRP reduction (Δ = −3.7 ± 1.5 mg/L vs. −1.9 ± 1.3 mg/L; *P* < 0.001).

**Table 3:** Comparison of inflammatory markers.

Parameter	Control group (*n* = 124)	Study group (*n* = 124)	Between-group	*P*-value
IL-6 (pg/mL)				
Baseline	11.9 ± 3.8	12.1 ± 3.7		0.676
12 months	8.5 ± 2.9	6.0 ± 2.3		<0.001
Change (Δ)	−3.4 ± 2.1	−6.1 ± 2.5	*t* = 9.167	<0.001
Within-group *P*	<0.001	<0.001		
TNF-α (pg/mL)				
Baseline	18.5 ± 4.8	18.8 ± 5.0		0.636
12 months	14.2 ± 3.9	10.7 ± 3.2		<0.001
Change (Δ)	−4.3 ± 2.8	−8.1 ± 3.2	*t* = 9.967	<0.001
Within-group *P*	<0.001	<0.001		
hs-CRP (mg/L)				
Baseline	6.5 ± 2.2	6.7 ± 2.4		0.498
12 months	4.6 ± 1.9	3.0 ± 1.5		<0.001
Change (Δ)	−1.9 ± 1.3	−3.7 ± 1.5	*t* = 10.063	<0.001
Within-group *P*	<0.001	<0.001		

Data are presented as mean ± standard deviation. Δ indicates absolute change from baseline to 12 months.

To examine whether the positive correlation between ΔD-dimer and Δhs-CRP observed in the study group (*r* = 0.48, 95% CI: 0.34–0.60, *P* < 0.001) was consistent across different patient subsets, we performed stratified analyses according to Markis classification and baseline D-dimer level.

Among patients with diffuse ectasia (Markis type I/II, *n* = 52 in the study group), the correlation was strong (*r* = 0.61, 95% CI: 0.40–0.76, *P* < 0.001), whereas in those with localized ectasia (Markis type III/IV, *n* = 72), the correlation was modest (*r* = 0.31, 95% CI: 0.09–0.50, *P* = 0.007). The difference between these two correlation coefficients was statistically significant (Fisher's *z* = 2.14, *P* = 0.032).

Similarly, when stratified by baseline D-dimer level, patients with high D-dimer (≥0.8 mg/L, *n* = 61) exhibited a stronger correlation (*r* = 0.59, 95% CI: 0.39–0.74, *P* < 0.001) compared with those with low D-dimer (<0.8 mg/L, *n* = 63), in whom the correlation was weaker and non-significant (*r* = 0.21, 95% CI: −0.04–0.44, *P* = 0.10). The between-subgroup difference was significant (Fisher's *z* = 2.31, *P* = 0.021).

These findings suggest that the “thrombo-inflammatory” coupling effect of rivaroxaban combination therapy is particularly pronounced in patients with diffuse ectasia and higher baseline thrombotic burden, aligning with the subgroup analysis of clinical outcomes.

Notably, correlation analysis revealed a significant positive association between the magnitude of D-dimer reduction and hs-CRP reduction in the study group (Pearson's *r* = 0.48, 95% CI: 0.34–0.60, *P* < 0.001), indicating that patients with greater antithrombotic response also experienced more pronounced anti-inflammatory effects. This correlation was substantially weaker and non-significant in the control group (*r* = 0.15, 95% CI: −0.03 to 0.32, *P* = 0.098). The between-group difference in correlation coefficients was statistically significant (Fisher's *z* = 2.87, *P* = 0.004), suggesting that rivaroxaban combination therapy may disrupt the pathological coupling between coagulation activation and inflammatory response in CAE patients—a finding consistent with the “thrombo-inflammator*y* axis” hypothesis.

### 3.4 Reduction in myocardial injury markers

Both treatment strategies significantly reduced markers of myocardial injury after 12 months (Table 4). Cardiac troponin I decreased by 0.09 ± 0.04 ng/mL in the study group compared to 0.06 ± 0.03 ng/mL in controls (*P* < 0.001). NT-proBNP reduction was also significantly greater with combination therapy (Δ = −302.5 ± 128.4 pg/mL vs. −164.3 ± 102.7 pg/mL; *P* < 0.001).

**Table 4:** Comparison of myocardial injury markers.

Parameter	Control group (*n* = 124)	Study group (*n* = 124)	Between-group	*P*-value
cTnI (ng/mL)				
Baseline	0.15 ± 0.06	0.15 ± 0.06		1
12 months	0.09 ± 0.04	0.06 ± 0.03		<0.001
Change (Δ)	−0.06 ± 0.03	−0.09 ± 0.04	*t* = 6.708	<0.001
Within-group P	<0.001	<0.001		
NT-proBNP (pg/mL)				
Baseline	618.5 ± 188.3	623.7 ± 191.5		0.83
12 months	454.2 ± 163.8	321.2 ± 139.6		<0.001
Change (Δ)	−164.3 ± 102.7	−302.5 ± 128.4	*t* = 9.438	<0.001
Within-group P	<0.001	<0.001		
High D-dimer subgroup (≥0.8 mg/L)				
*n*	58	61		
NT-proBNP change (Δ, pg/mL)	−181.5 ± 118.7	−358.6 ± 142.3	t = 7.376	<0.001

Data are presented as mean ± standard deviation. Δ indicates absolute change from baseline to 12 months.

When stratified by baseline thrombotic burden (D-dimer ≥0.8 mg/L), the benefit of combination therapy on NT-proBNP reduction was particularly pronounced. In patients with high baseline D-dimer, the NT-proBNP reduction in the study group was nearly twice that of the control group (Δ = −358.6 ± 142.3 pg/mL vs. −181.5 ± 118.7 pg/mL; *P* < 0.001), suggesting that patients with more severe hypercoagulability derive greater myocardial benefit from intensified anticoagulation.

### 3.5 Clinical outcomes: major adverse cardiovascular events and survival analysis

During the follow-up period, the study group demonstrated significantly lower incidence of major adverse cardiovascular events compared to the control group (Table 5). Total MACE occurred in 10 patients (8.1%) in the study group vs. 27 patients (21.8%) in the control group (HR = 0.34, 95% CI: 0.19–0.62, *P* < 0.001). The absolute risk reduction was 13.7%, corresponding to a number needed to treat (NNT) of 7.3 to prevent one MACE over 36 months.

**Table 5:** Comparison of major adverse cardiovascular events.

Outcome	Control group (*n* = 124)	Study group (*n* = 124)	HR (95% CI)	*P*-value
Cardiac death	0 (0.0)	0 (0.0)	–	–
Non-fatal myocardial infarction	6 (4.8)	2 (1.6)	0.33 (0.07–1.64)	0.154
Non-fatal ischemic stroke	4 (3.2)	2 (1.6)	0.50 (0.09–2.73)	0.415
Rehospitalization for myocardial ischemia	11 (8.9)	4 (3.2)	0.36 (0.11–1.13)	0.07
Repeat revascularization	6 (4.8)	2 (1.6)	0.33 (0.07–1.64)	0.154
Total MACE	27 (21.8)	10 (8.1)	0.34 (0.19–0.62)	<0.001

Data are presented as *n* (%).

HR, hazard ratio; CI, confidence interval; MACE, major adverse cardiovascular events.

**Figure 3:**
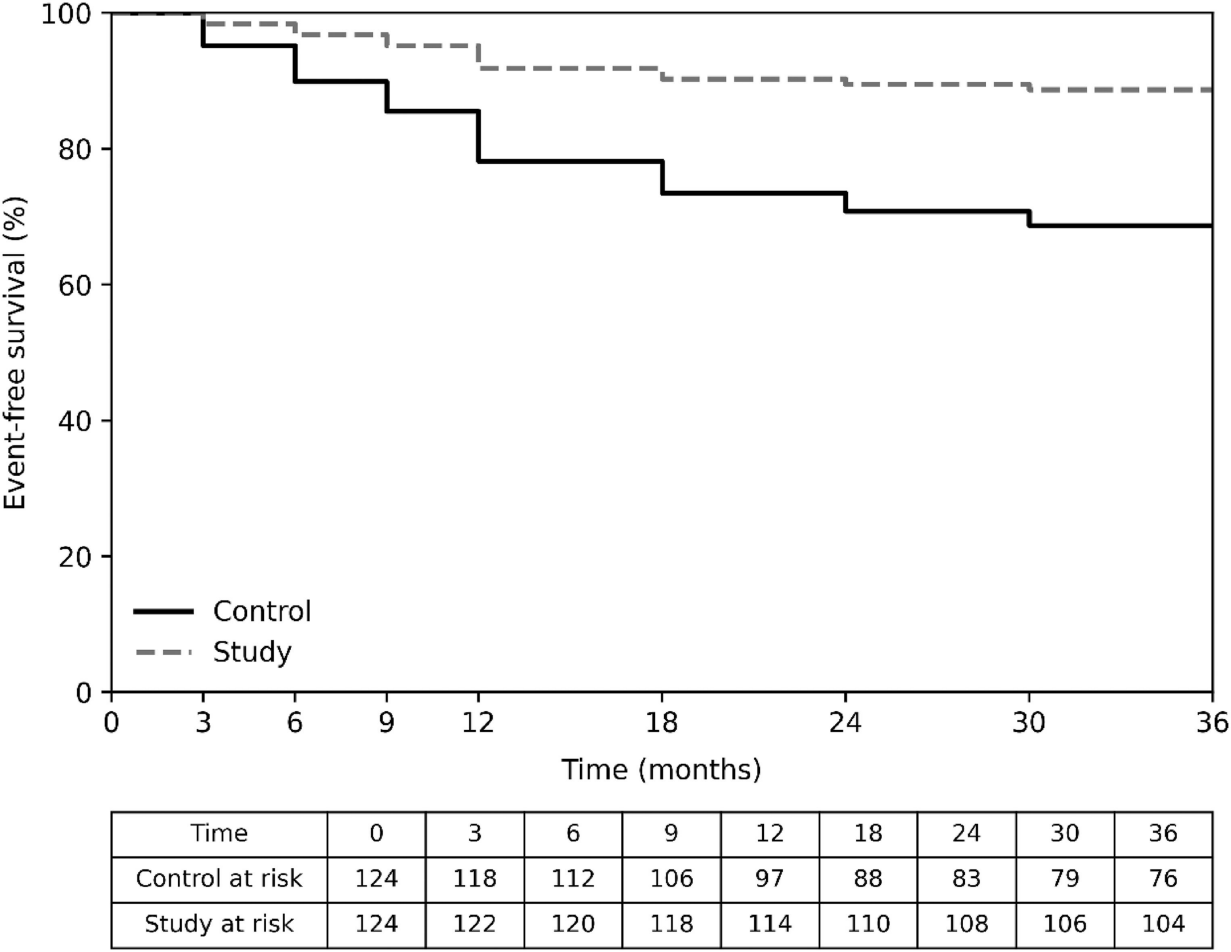
Kaplan-Meier curves for freedom from MACE.

Kaplan–Meier survival analysis showed early divergence of the event curves, with the study group maintaining consistently higher event-free survival throughout the follow-up period (Log-rank *P* = 0.002; Figure 3). The separation became apparent as early as 3 months post-treatment, suggesting a relatively rapid clinical benefit from combination therapy.

### 3.6 Subgroup analysis of treatment effect on MACE

**Figure 4:**
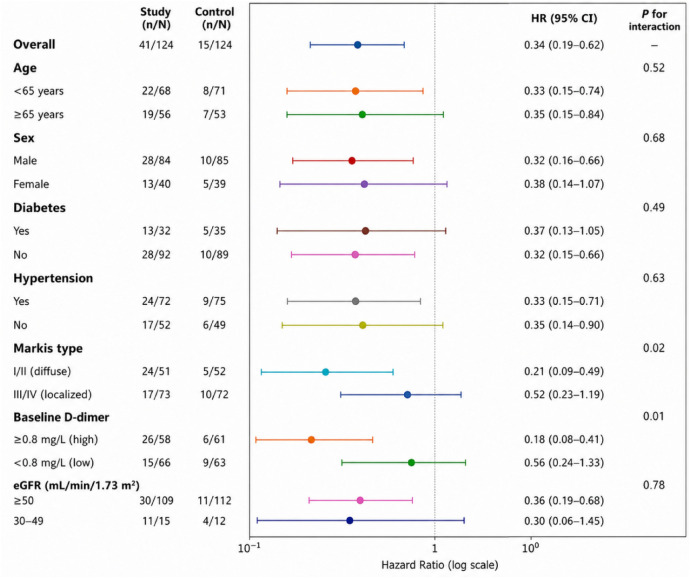
Forest plot of subgroup analysis for MACE.

To identify patient populations that might derive greater benefit from combination therapy, we performed prespecified subgroup analyses. The forest plot in Figure 4 shows the hazard ratios for MACE across various subgroups.

The benefit of rivaroxaban combination therapy was consistent across most subgroups. Significant heterogeneity was observed for Markis classification and baseline D-dimer levels. Patients with diffuse ectasia (Markis type I/II) derived greater benefit (HR = 0.21, 95% CI: 0.09–0.49) compared to those with localized ectasia (Markis type III/IV: HR = 0.52, 95% CI: 0.23–1.19; interaction *P* = 0.02). Similarly, patients with high baseline D-dimer (≥0.8 mg/L) showed more pronounced risk reduction (HR = 0.18, 95% CI: 0.08–0.41) than those with lower D-dimer (HR = 0.56, 95% CI: 0.24–1.33; interaction *P* = 0.01). There was no significant interaction for age, sex, diabetes, or hypertension, indicating that the treatment effect is broadly applicable but particularly enhanced in patients with higher thrombotic burden.

We further explored whether baseline renal function (eGFR ≥50 vs. 30–49 mL/min/1.73 m^2^) modified the treatment effect on 36-month MACE. Among patients with eGFR ≥50 (*n* = 221), the HR for MACE with study vs. control was 0.36 (95% CI: 0.19–0.68). In the smaller subgroup with eGFR 30–49 (*n* = 27), the HR was 0.30 (95% CI: 0.06–1.45), with no significant interaction (*P* for interaction = 0.78). However, the small number of patients with eGFR 30–49 (*n* = 27, 10.9% of the matched cohort) limits the statistical power to detect a true interaction; therefore, these results should be interpreted as exploratory and hypothesis-generating.

Regarding safety, major bleeding (BARC type 3) occurred in one patient with eGFR ≥50 in the study group and one patient with eGFR 30–49 in the control group. No excess bleeding was observed in the reduced eGFR subgroup on rivaroxaban. These findings should be interpreted with caution due to the limited sample size in the eGFR 30–49 stratum.

### 3.7 Safety outcomes and net clinical benefit

During the 36-month follow-up, no fatal bleeding events occurred in either group. Bleeding events were classified according to the BARC criteria. The overall incidence of any bleeding was higher in the study group (22.6% vs. 15.3%), but the difference did not reach statistical significance (*P* = 0.15). Most bleeding events were BARC type 1 (minor) such as gingival bleeding, epistaxis, or subcutaneous ecchymosis, which resolved spontaneously or with local measures. BARC type 2 bleeding (clinically relevant non-major) occurred in 4.8% of study patients and 2.4% of controls (*P* = 0.31). BARC type 3 (major) bleeding was rare and comparable between groups (0.8% vs. 0.8%, *P* = 1.00). No BARC type 4 or 5 bleeding was observed (Table 6).

**Table 6:** Bleeding events according to BARC criteria.

Bleeding type	Control group (*n* = 124)	Study group (*n* = 124)	*P*-value
BARC type 1 (minor)	16 (12.9)	21 (16.9)	0.38
Gingival bleeding	5 (4.0)	7 (5.6)	0.56
Epistaxis	3 (2.4)	5 (4.0)	0.47
Subcutaneous ecchymosis	6 (4.8)	7 (5.6)	0.78
Other minor	2 (1.6)	2 (1.6)	1
BARC type 2 (clinically relevant non-major)	3 (2.4)	6 (4.8)	0.31
Gastrointestinal	1 (0.8)	3 (2.4)	0.31
Hematuria	1 (0.8)	2 (1.6)	0.56
Other	1 (0.8)	1 (0.8)	1
BARC type 3 (major)	1 (0.8)	1 (0.8)	1
Requiring transfusion	1 (0.8)	1 (0.8)	1
Total bleeding events	19 (15.3)	28 (22.6)	0.15
Discontinuation due to bleeding, *n* (%)	2 (1.6)	5 (4.0)	0.45
Permanent discontinuation	1 (0.8)	3 (2.4)	0.62
Temporary discontinuation	1 (0.8)	2 (1.6)	1.00

Data are presented as *n* (%). BARC, Bleeding Academic Research Consortium. Temporary discontinuation was defined as cessation of the offending agent for >7 days directly attributable to a bleeding event, with subsequent restart. Duration ranged from 8 to 21 days in the study group and 10 to 14 days in the control group.

Among the 28 bleeding events in the study group, the median time to first bleeding was 5.2 months (IQR: 2.1–12.8 months). Eleven events (39.3%) occurred within the first 3 months of treatment, 9 (32.1%) between 3 and 12 months, and 8 (28.6%) after 12 months. In the control group (19 events), the median time to first bleeding was 7.6 months (IQR: 3.3–15.4 months), with 6 events (31.6%) occurring within 3 months, 7 (36.8%) between 3 and 12 months, and 6 (31.6%) after 12 months. The timing distribution did not differ significantly between groups (log-rank *P* = 0.34 for time to first bleeding).

For time-to-first-bleeding analysis, patients who died (*n* = 0) or were lost to follow-up (*n* = 0 by design) without experiencing a bleeding event were censored at their last follow-up date. All patients contributed complete follow-up data.

Treatment discontinuation due to bleeding occurred in 5 patients (4.0%) in the study group and 2 patients (1.6%) in the control group (*P* = 0.45). In the study group, rivaroxaban was permanently discontinued in 3 patients (one with BARC type 3a bleeding requiring transfusion, one with recurrent BARC type 2 gastrointestinal bleeding, and one with BARC type 2 hematuria) and temporarily withheld (<7 days) in 2 patients (both BARC type 2). No patient in the study group discontinued antiplatelet therapy alone. In the control group, antiplatelet therapy was discontinued permanently in 1 patient (aspirin-related gastric ulcer with BARC type 2 bleeding) and temporarily in 1 patient (BARC type 1 epistaxis with recurrent episodes).

**Figure 5:**
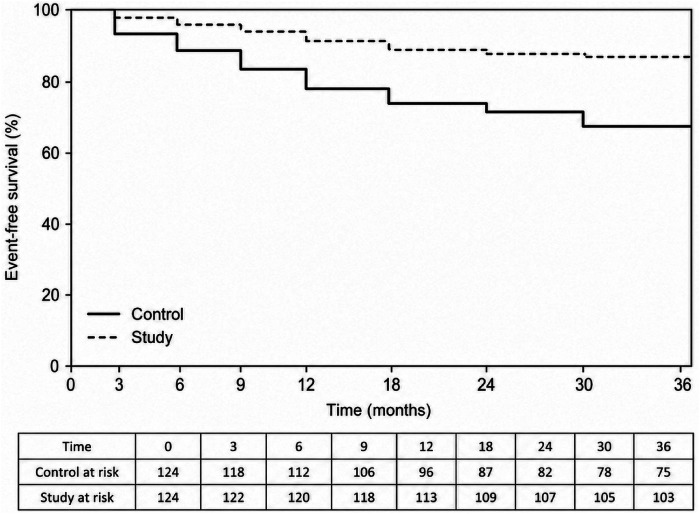
Net clinical benefit.

To assess the overall benefit-risk balance, we calculated the net clinical benefit, defined as the composite of MACE plus BARC type 3 (major) bleeding. The study group had a significantly lower net clinical event rate compared to the control group (8.9% vs. 22.6%; HR = 0.37, 95% CI: 0.19–0.74, *P* = 0.003), confirming that the reduction in ischemic events outweighs the modest, non-significant increase in bleeding risk (Figure 5).

### 3.8 Sensitivity analyses

**Figure 6:**
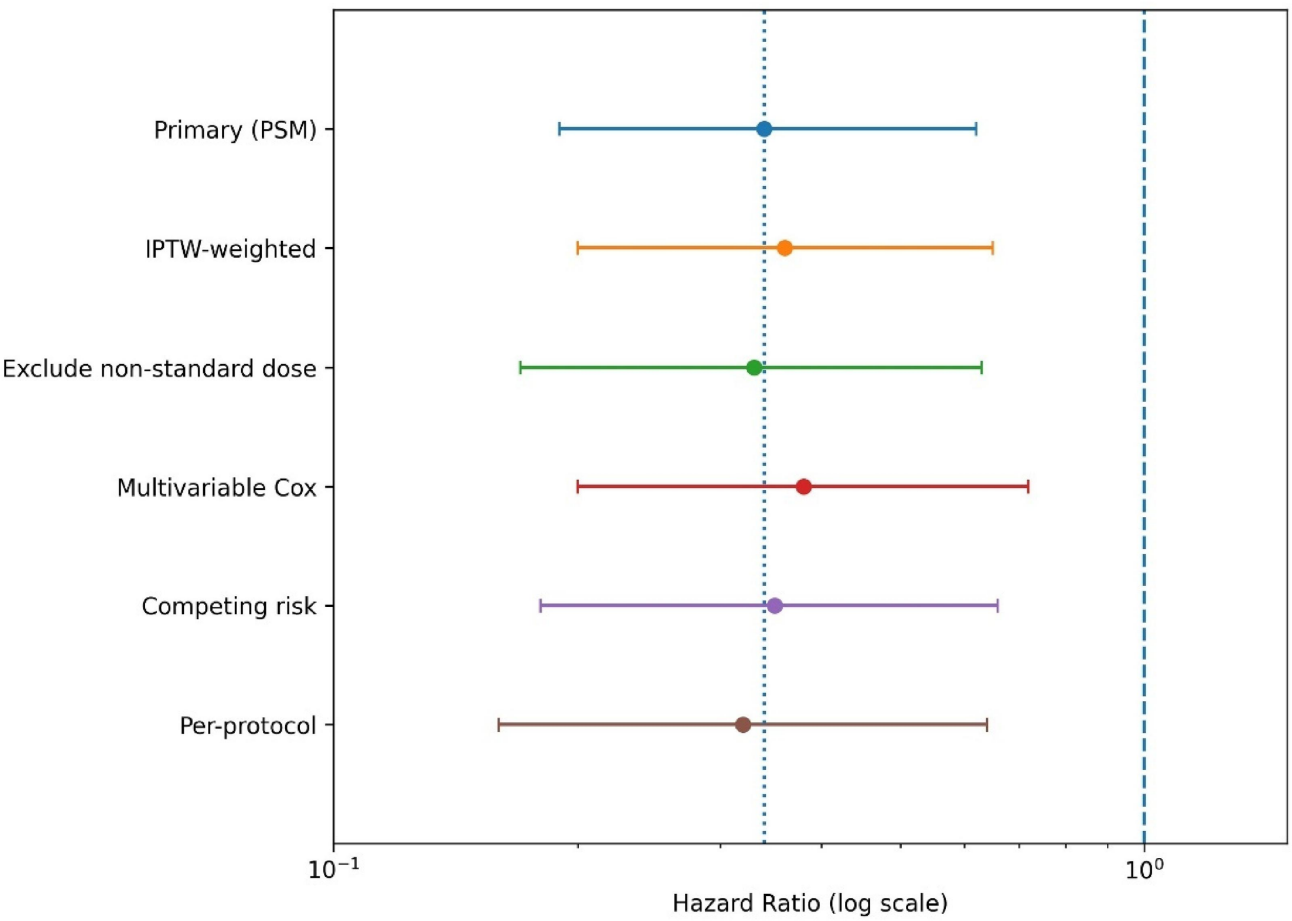
Forest plot of sensitivity analyses for 36-month MACE.

To assess the robustness of our primary findings, we conducted several prespecified sensitivity analyses (Figure 6). As an alternative to propensity score matching, we performed inverse probability of treatment weighting using the same propensity score model, which achieved excellent covariate balance (all standardized mean differences <0.05). The IPTW-adjusted Cox model confirmed a consistent treatment effect on 36-month MACE (HR = 0.36, 95% CI: 0.20–0.65, *P* < 0.001), nearly identical to the primary matched analysis (HR = 0.34). To address potential confounding by dose variation in the study group, we performed a sensitivity analysis excluding patients who deviated from the standard protocol-defined regimen. Specifically, we excluded 11 patients in the study group who received rivaroxaban 10 mg once daily instead of the standard 2.5 mg twice daily regimen, and 7 patients in the control group who deviated from the prescribed single antiplatelet therapy (e.g., receiving an alternative antiplatelet agent not specified in the protocol or with documented non-adherence affecting dose/agent beyond acceptable limits). In the remaining cohort (control: *n* = 117; study: *n* = 113), the treatment effect remained robust (HR for 36-month MACE = 0.33, 95% CI: 0.17–0.63, *P* < 0.001). This analysis confirms that the primary findings are not driven by non-standard rivaroxaban dosing or by minor protocol deviations in the control group. Multivariable Cox regression adjusting for all baseline covariates in the original unmatched cohort (*n* = 312) demonstrated that rivaroxaban combination therapy remained independently associated with reduced MACE risk (HR = 0.38, 95% CI: 0.20–0.72, *P* = 0.003), consistent with the primary analysis. Considering that non-cardiovascular death (2 events in control group, 1 in study group) may act as a competing risk for MACE, we performed Fine-Gray subdistribution hazard regression, which yielded a significant subdistribution hazard ratio for MACE (sHR = 0.35, 95% CI: 0.18–0.66, *P* < 0.001), indicating that the benefit of combination therapy was not biased by competing mortality. Finally, a per-protocol analysis censoring patients who discontinued rivaroxaban or antiplatelet therapy for >30 days during follow-up (12 patients in study group, 8 in control group) produced consistent results (HR = 0.32, 95% CI: 0.16–0.64, *P* < 0.001). Collectively, these sensitivity analyses confirm the robustness of our primary findings and support the conclusion that adding rivaroxaban to single antiplatelet therapy significantly reduces long-term MACE risk in patients with CAE.

### 3.9 Risk prediction model: MACE risk prediction based on baseline D-dimer, markis classification, and treatment regimen

Propensity score matching, while useful for treatment effect estimation, alters the natural distribution of covariates by design. Therefore, risk estimates derived from the matched cohort may not be generalizable to the original unmatched CAE population. To assess the robustness of the model, we also fitted the same model in the unmatched cohort (*n* = 312); the C-index was 0.75 (95% CI: 0.68–0.82), and the direction and magnitude of associations were similar. Nonetheless, external validation in an independent cohort is essential before clinical application.

To facilitate clinical decision-making and identify high-risk patients most likely to benefit from rivaroxaban combination therapy, we developed a simplified risk prediction model using Cox proportional hazards regression. The model was developed in the propensity score-matched cohort (*n* = 248) with 36-month MACE as the outcome, incorporating three readily available clinical variables: baseline D-dimer level (continuous variable, natural log-transformed), Markis classification (Type I/II diffuse ectasia vs. Type III/IV localized ectasia), and treatment group (study group vs. control group). Model discrimination was assessed using Harrell's C-index, calibration was evaluated using calibration plots and the Hosmer-Lemeshow test, and internal validation was performed using bootstrapping (500 resamples). Additionally, decision curve analysis was employed to evaluate the net clinical benefit of the model, comparing it against a simplified model based solely on treatment group, as well as “treat-all” and “treat-none” strategies.

Model results (Table 7) showed that all three variables were independent predictors of MACE. Each one-unit increase in log-transformed baseline D-dimer was associated with a 2.53-fold increase in MACE risk (HR = 2.53, 95% CI: 1.62–3.95, *P* < 0.001); The prediction model identified Markis type I/II as an independent predictor of higher MACE risk (HR = 1.89, 95% CI: 1.18–3.03, *P* = 0.008), indicating that patients with diffuse ectasia have an 89% higher baseline risk compared to those with localized ectasia, after adjusting for treatment and D-dimer; and patients in the study group had a 65% lower MACE risk compared to the control group (HR = 0.35, 95% CI: 0.19–0.64, *P* < 0.001). The model demonstrated good discrimination with a C-index of 0.78 (95%CI 0.72–0.84). After bootstrap optimism correction (500 resamples), the optimism-adjusted C-index was 0.76 (95% CI: 0.70–0.82), indicating that the model's discrimination remains acceptable but is more modest than the apparent C-index suggests. The calibration slope after bootstrapping was 0.88 (ideal value = 1), with a slight tendency toward overfitting.

**Table 7:** Multivariable cox regression prediction model.

Variable	β Coefficient	HR (95% CI)	*P*-value
Baseline log(D-dimer)	0.93	2.53 (1.62–3.95)	<0.001
Markis type I/II (vs. III/IV)	0.64	1.89 (1.18–3.03)	0.008
Study group (vs. Control group)	−1.05	0.35 (0.19–0.64)	<0.001

D-dimer was natural log-transformed prior to model inclusion.

HR, hazard ratio; CI, confidence interval.

For clinical interpretability, a 0.5 mg/L increase in raw D-dimer (from 0.5 to 1.0 mg/L) corresponds to an HR of approximately 1.45 (95% CI: 1.23–1.71), derived from the log-transformed model. This estimate assumes constant proportional hazards across the range of D-dimer values.

**Figure 7:**
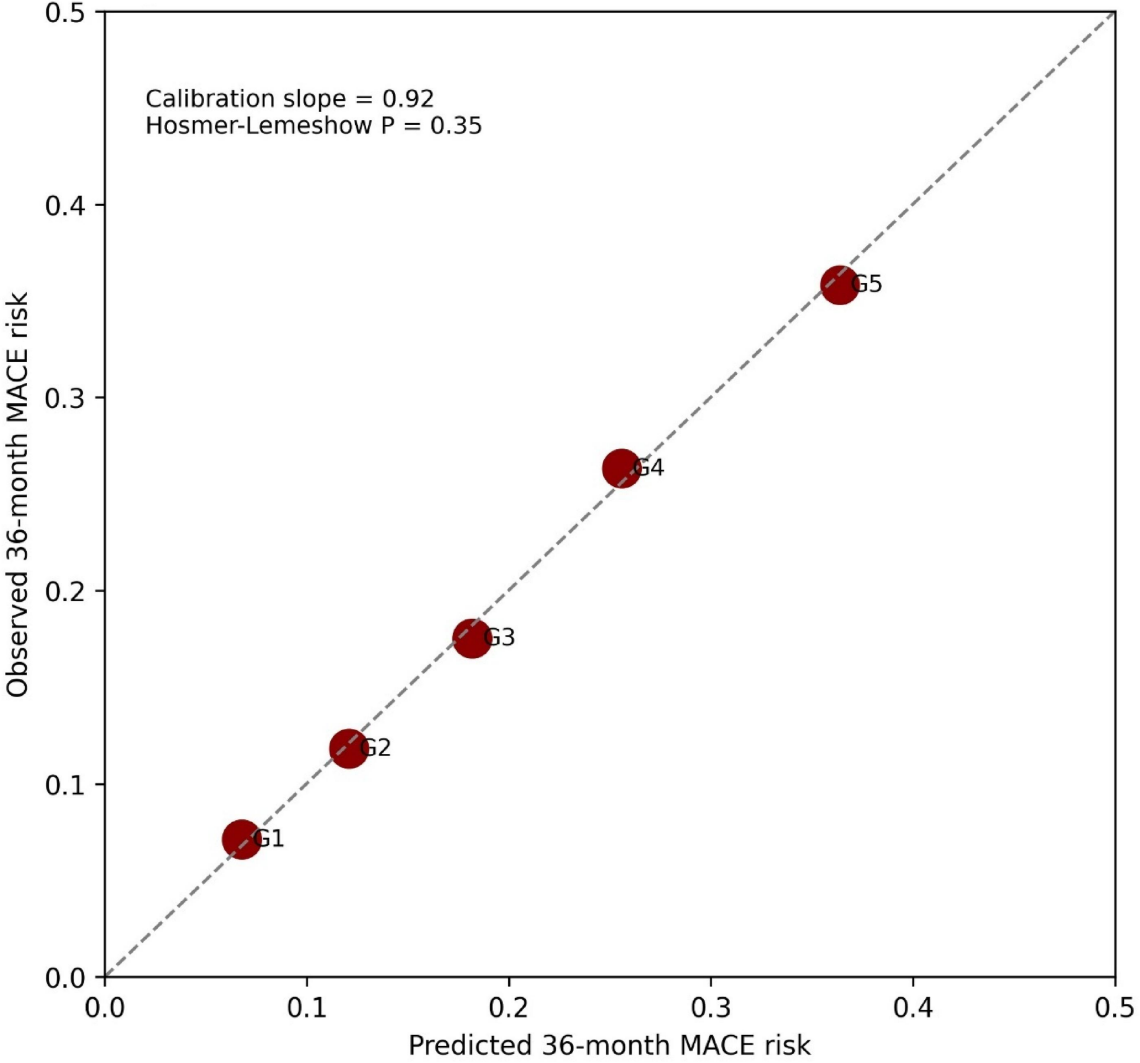
Calibration plot of the 36-month MACE prediction model.

Regarding model calibration (Figure 7), patients were grouped into quintiles based on predicted risk, and the mean predicted probability for each quintile was plotted against the observed probability estimated by the Kaplan–Meier method. The predicted and observed probabilities were evenly distributed around the 45-degree diagonal line, with a Hosmer-Lemeshow test *P*-value of 0.35 and a calibration slope of 0.92 (ideal value = 1), indicating good calibration.

**Figure 8:**
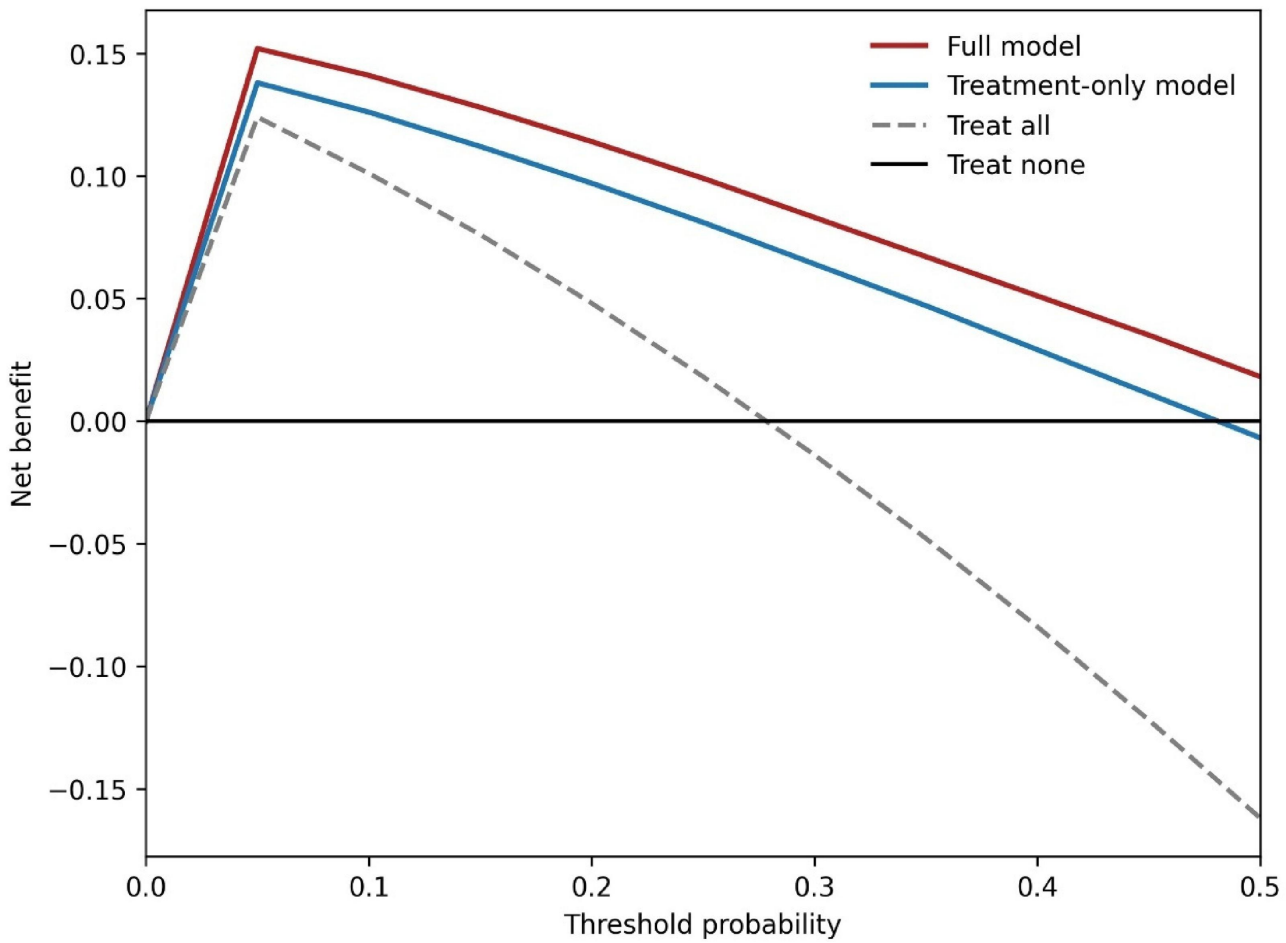
Decision curve analysis for the 36-month MACE prediction model.

For clinical utility assessment, decision curve analysis (Figure 8) compared the net benefit of our prediction model, a simplified model based solely on treatment group, the “treat-all” strategy, and the “treat-none” strategy across different threshold probabilities. Our model yielded the highest net benefit within the threshold probability range of 5%–35%, substantially outperforming the other strategies and demonstrating excellent clinical practical value.

**Figure 9:**
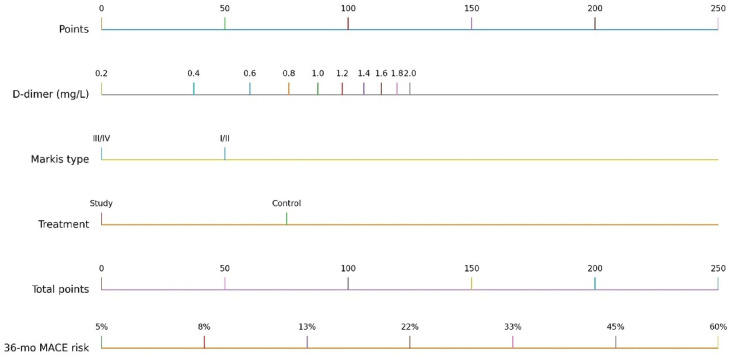
Nomogram for predicting 36-month MACE risk.

To facilitate clinical application, we constructed a nomogram based on the above model (Figure 9). The nomogram transforms the complex regression equation into an intuitive graphical tool, enabling direct reading of the predicted 36-month MACE probability based on a patient's D-dimer level, Markis classification, and treatment group.

## 4 Discussion

This 36-month follow-up study systematically evaluated the long-term efficacy and safety of single antiplatelet therapy combined with rivaroxaban in patients with CAE. The main findings were as follows: the combination therapy group exhibited significantly greater improvements in thrombotic, inflammatory, and myocardial injury markers, along with a 66% reduction in the risk of 36-month major adverse cardiovascular events (HR = 0.34, 95%CI 0.19–0.62, *P* < 0.001), corresponding to an absolute risk reduction of 13.7% and a number needed to treat of 7.3. Patients with Markis type I/II diffuse ectasia and elevated baseline D-dimer levels derived particularly pronounced benefits. Regarding safety, combination therapy did not significantly increase the risk of major bleeding, and the net clinical benefit at 36 months was clear, with multiple sensitivity analyses confirming the robustness of these findings. These observations provide preliminary evidence-based reference for long-term antithrombotic strategies in patients with CAE.

As a distinct coronary pathology, CAE is characterized by abnormal luminal dilation, slow blood flow, and vortex formation, leading to blood stasis (18, 19). This prothrombotic and proinflammatory environment predisposes to coagulation activation and thrombus deposition (20, 21). In our study, the 36-month MACE rate of 21.8% in the control group confirms that CAE patients face substantial long-term risk. The higher event rate in our CAE cohort (21.8%) suggests that CAE may confer additional thrombotic risk beyond that of typical atherosclerotic disease. And simply adhering to conventional antiplatelet strategies used for coronary artery disease may be insufficient to address their thrombotic risk. The 36-month MACE rate of 21.8% in our control group is comparable to the 16.3% rate reported by Gurgoglione et al. (12) in CAE patients with MINOCA over a similar follow-up period, confirming that CAE patients face substantial long-term cardiovascular risk even in the absence of severe obstructive lesions. Our results show that adding rivaroxaban to single antiplatelet therapy improved thrombotic and coagulation parameters in CAE patients. After 12 months, the combination group had greater reductions in D-dimer and fibrinogen and greater prolongations of PT and APTT, indicating more effective inhibition of thrombin generation and fibrin formation. In CAE, slow flow and low shear stress lead to platelet-coagulation activation (22, 23). Single antiplatelet therapy targets only platelet aggregation, leaving thrombin-driven amplification unchecked. Rivaroxaban directly inhibits factor Xa, blocking thrombin at its source and reducing thrombotic burden (24). This dual-pathway inhibition strategy has been validated in stable coronary artery disease and peripheral artery disease through the COMPASS trial (9, 11, 25, 26). While direct evidence for its application to CAE is lacking, the pathophysiological rationale is compelling: CAE is characterized by abnormal luminal dilation, slow flow, and vortex formation, leading to a prothrombotic state driven by both platelet aggregation and thrombin generation. Therefore, the application of dual-pathway inhibition to CAE remains a hypothesis based on pathophysiological plausibility rather than established evidence. Our study aimed to provide preliminary observational data to test this hypothesis in the CAE population. The observed improvements in D-dimer and coagulation parameters in the combination group are consistent with this mechanistic framework, but causality cannot be inferred from this observational design.

Inflammation and coagulation are closely intertwined in CAE. Chronic inflammatory infiltration and cytokine release (IL-6, TNF-α) promote tissue factor expression and coagulation activity, while thrombin in turn amplifies inflammation via protease-activated receptors, creating a self-reinforcing cycle (27, 28). Factor Xa can mediate non-hemostatic cellular effects through PAR-1 and PAR-2 receptors, including cardiomyocyte hypertrophy, fibroblast proliferation, and inflammatory cytokine expression (29). In a pressure-overload mouse model, low-dose rivaroxaban attenuated cardiac inflammation, hypertrophy, and fibrosis, and improved diastolic function (29). In our study, the decline in inflammatory markers was more pronounced in the rivaroxaban combination group compared with the control group. While this observation is consistent with the hypothesis that intensified anticoagulation may interrupt the coagulation-inflammation cycle, causality cannot be inferred from this observational study. The possibility of residual confounding (e.g., patients with higher baseline inflammation may have been preferentially prescribed rivaroxaban, or unmeasured lifestyle factors) cannot be excluded. These findings should be considered hypothesis-generating. The emerging concept of “immunothrombosis” posits that anticoagulation not only prevents thrombosis but may also attenuate inflammation by inhibiting thrombin-mediated pathways (30). Factor Xa inhibitors have been shown to reduce endothelial inflammatory cytokines and oxidative stress (31). The concurrent reductions in IL-6, TNF-α, and hs-CRP observed in our study are consistent with this mechanism, suggesting that combined anticoagulation may improve the vascular microenvironment. More importantly, the significant positive correlation between ΔD-dimer and Δhs-CRP in the study group, which was absent in the control group, with a significant between-group difference in correlation coefficients (*P* = 0.004), provides observational evidence consistent with the hypothesis that rivaroxaban may disrupt the coagulation-inflammation feedback loop. This correlation is consistent with—but does not prove—the hypothesis that rivaroxaban may disrupt the coagulation-inflammation feedback loop. An alternative explanation is that rivaroxaban, as a more potent antithrombotic agent, produces greater reductions in thrombotic burden (reflected by D-dimer), which in turn may secondarily reduce inflammation; the observed correlation could merely reflect the co-variation of two treatment responses rather than a direct causal mechanism. The temporal relationship between changes in D-dimer and hs-CRP could not be established due to the absence of serial biomarker measurements. Therefore, this finding should be considered an intriguing hypothesis derived from correlational data, warranting confirmation in prospective studies with repeated biomarker sampling and mechanistic investigations.

In our study, the combination therapy group showed significantly greater reductions in cTnI and NT-proBNP. CAE patients are prone to subclinical myocardial ischemia and microcirculatory disturbances due to slow flow and microthrombus formation, which may lead to cardiomyocyte injury and increased wall stress (32). By reducing microthrombus burden and improving hemorheology, rivaroxaban may lower microcirculatory resistance and enhance myocardial oxygen supply, thereby decreasing ventricular wall tension (33). These findings align with prior evidence that intensified antithrombotic therapy improves microcirculatory perfusion (34–36). Notably, in patients with high baseline D-dimer (≥0.8 mg/L), NT-proBNP reduction was nearly twice that of controls, suggesting greater myocardial protection in those with higher thrombotic burden—consistent with our clinical subgroup analyses.

Most importantly, the 36-month follow-up results showed that the overall incidence of MACE was significantly lower in the study group compared with the control group, with an absolute risk reduction of 13.7%. The number needed to treat (NNT) over 36 months was 7.3, derived from an absolute risk reduction of 13.7% (21.8% − 8.1%). It is important to note that the NNT is population-specific; in cohorts with lower baseline MACE risk, the NNT would be higher, whereas in higher-risk patients, the NNT could be even lower. Therefore, this NNT should not be directly generalized to all CAE populations without considering individual baseline risk. Kaplan–Meier curves showed significantly higher event-free survival in the combination group from month 3 through 36 (log-rank *P* = 0.002), indicating early and sustained clinical benefit. Although some individual endpoints were not statistically significant, the overall trend was consistent, particularly for rehospitalization for ischemia and repeat revascularization. Given the slow-flow nature of CAE, these patients may be more sensitive to anticoagulation than typical atherosclerotic populations.

Patients with Markis I/II (diffuse) ectasia derived greater benefit than those with localized ectasia (interaction *P* = 0.02), as did patients with baseline D-dimer ≥0.8 mg/L (interaction *P* = 0.01). Thus, diffuse ectasia and high thrombotic burden are key indicators for identifying patients most likely to benefit from intensified anticoagulation. No fatal bleeding occurred. Total bleeding did not differ significantly between groups. Minor bleeding was slightly more frequent in the combination group but was mild and manageable. BARC type 3 major bleeding was rare and comparable between groups (0.8% each, *P* = 1.00), indicating acceptable long-term safety with appropriate patient selection. In our matched cohort, the mean HAS-BLED score was 1.8 ± 0.7 in the control group and 1.9 ± 0.7 in the study group, indicating predominantly low bleeding risk. Net clinical benefit (MACE + major bleeding) was significantly higher in the combination group, confirming that the reduction in ischemic events outweighs the modest bleeding risk. The high MACE rate in the control group (21.8%) reflects the elevated risk profile of our CAE cohort.

Multiple sensitivity analyses (IPTW, exclusion of non-standard doses, multivariable Cox, competing risk, per-protocol) all yielded results consistent with the primary analysis, confirming the robustness of our findings.

The risk prediction model based on D-dimer, Markis classification, and treatment regimen showed good discrimination (C-index 0.78) and calibration. Decision curve analysis demonstrated superior net benefit within the 5%–35% threshold range. The model uses readily available variables, offers an intuitive nomogram for outpatient use, and simultaneously assesses baseline risk and anticipated treatment benefit—consistent with the finding that high-risk patients derive greater benefit. This study establishes the first exploratory prediction model specifically for the CAE population. However, this model was derived from a single-center cohort and has not been externally validated. Its performance in other CAE populations remains unknown; therefore, it should be considered hypothesis-generating and used cautiously in clinical decision-making until validated in independent cohorts.

This study provides 36-month observational data for the CAE population, a group lacking guideline-directed management, suggesting that in selected patients, single antiplatelet therapy combined with low-dose rivaroxaban may be associated with lower MACE risk, particularly in those with Markis type I/II diffuse ectasia or elevated baseline D-dimer levels. The number needed to treat at 36 months was 7.3, corresponding to an annualized NNT of approximately 22 (based on an annualized absolute risk reduction of 4.6% per year). The lower NNT in our CAE population suggests a more pronounced treatment effect, consistent with the higher baseline thrombotic risk of CAE patients. Under the premise of rigorous bleeding risk assessment (HAS-BLED score <3, normal renal function), the bleeding risk of combination therapy appears manageable with a clear net clinical benefit. These findings offer preliminary reference for individualized antithrombotic decision-making in patients with CAE.

Our finding that rivaroxaban plus single antiplatelet therapy was associated with a 66% reduction in MACE risk (HR = 0.34, 95% CI: 0.19–0.62) contrasts with the conclusions of a recent network meta-analysis, which ranked DAPT as the most effective strategy for CAE. This discrepancy may be explained by differences in study populations (our cohort was predominantly at low bleeding risk and included patients with diffuse ectasia who may derive greater benefit from anticoagulation), treatment regimens (we evaluated low-dose rivaroxaban plus single antiplatelet, whereas the network meta-analysis primarily assessed anticoagulant monotherapy or DAPT), and the observational nature of our study. Prospective randomized comparisons of DAPT vs. rivaroxaban plus single antiplatelet in CAE are needed to resolve this uncertainty.

This study has several limitations. First, its single-center retrospective design, although adjusted with propensity score matching, cannot completely exclude selection bias and unmeasured confounding. While most variables achieved SMD <0.1, PT had an SMD of 0.11, marginally exceeding the prespecified threshold. This indicates a minor residual imbalance in this coagulation parameter, which should be considered a potential confounder in subsequent analyses. However, given that PT was not included in the propensity score model (by design, as it was measured post-admission and could lie on the causal pathway), and the imbalance is modest, we do not expect it to materially affect the primary treatment effect estimate. Nonetheless, this minor imbalance is acknowledged as a limitation. Second, the small sample size resulted in wide confidence intervals for key subgroups: the clopidogrel subgroup (*n* = 30/28, HR = 0.31, 95% CI: 0.08–1.25) and the eGFR 30–49 subgroup (*n* = 27, HR = 0.30, 95% CI: 0.06–1.45). The observed power for detecting a 40% relative risk reduction in these subgroups was <30%; thus, these findings are hypothesis-generating only. Third, rivaroxaban dosing was not uniform: 91.1% received 2.5 mg bid (COMPASS-tested), while 11 patients (8.9%) received 10 mg once daily, a regimen not evaluated in COMPASS with different pharmacokinetics. However, sensitivity analysis excluding these 11 patients yielded consistent results (HR = 0.33, 95% CI: 0.17–0.63). Fourth, the 36-month follow-up is insufficient for lifetime risk assessment. Fifth, the absence of repeated laboratory measurements (only baseline and 12 months) means that the observed biomarker improvements could be partially attributable to regression to the mean rather than a true treatment effect, especially without a placebo control. Sixth, we did not perform quantitative bias analysis or use instrumental variables to adjust for unmeasured confounders (e.g., physician preference, socioeconomic status). Seventh, the proportion of patients with moderate renal impairment was small (10.9%). In this subgroup (control: 2/13, 15.4%; study: 1/14, 7.1%; HR = 0.30, 95% CI: 0.06–1.45), the power to detect a 50% relative risk reduction was <20%; the lack of statistical significance (*P* for interaction = 0.78) does not rule out a clinically meaningful difference. Eighth, the risk prediction model was derived and internally validated in the same matched cohort (*n* = 248). While we used bootstrap optimism correction to adjust for overfitting, this does not substitute for external validation in an independent CAE cohort. The model's performance, including the C-index of 0.76 after correction, may not generalize to other populations with different case mix, referral patterns, or treatment protocols. Until external validation is performed, the nomogram should be considered exploratory and hypothesis-generating, not a clinical decision tool. Moreover, we acknowledge that pooling aspirin and clopidogrel in the background antiplatelet regimen introduces clinically relevant heterogeneity. We did not stratify or match by specific antiplatelet agent because the sample size (*n* = 28 and *n* = 30 in the clopidogrel subgroups after matching) was insufficient to support meaningful subgroup analyses or separate propensity score matching. Furthermore, the choice of antiplatelet agent (aspirin vs. clopidogrel) was predominantly determined by aspirin tolerance and gastrointestinal risk rather than by thrombotic or bleeding risk profile, reducing its likelihood of being a strong effect modifier in this cohort. Nevertheless, the possibility remains that the incremental benefit and bleeding risk of adding rivaroxaban could differ between aspirin- and clopidogrel-treated patients, and the underpowered exploratory analysis cannot resolve this uncertainty. This heterogeneity should be considered a primary limitation of the study, and the results should be interpreted as reflecting the average treatment effect across a mixed antiplatelet background, not as evidence for the combination with any specific agent. Future prospective, multicenter randomized controlled trials are warranted to confirm the efficacy and safety of low-dose rivaroxaban combined with single antiplatelet therapy in patients with CAE. Such trials should incorporate serial biomarker measurements (e.g., D-dimer, hs-CRP) to establish temporal causality and distinguish treatment effects from regression to the mean. To address the heterogeneity in treatment response, trials should stratify randomization by Markis classification (diffuse vs. localized) and baseline D-dimer levels. For patients with moderate renal impairment (eGFR 30–49 mL/min/1.73 m^2^), dedicated pharmacokinetic and safety studies are needed to determine optimal rivaroxaban dosing. Furthermore, external validation of the proposed risk prediction model in independent CAE cohorts is essential before clinical implementation. Finally, future research should employ quantitative bias analysis or instrumental variable methods to account for unmeasured confounders (e.g., physician preference, socioeconomic status) inherent in real-world studies.

## 5 Conclusion

In this retrospective cohort study of patients with coronary artery ectasia, low-dose rivaroxaban plus single antiplatelet therapy was associated with a lower 36-month MACE risk compared with single antiplatelet alone (8.1% vs. 21.8%; HR = 0.34, 95% CI: 0.19–0.62; absolute risk reduction 13.7%; NNT = 7.3), without a significant increase in major bleeding. The association was more pronounced in patients with diffuse ectasia and elevated D-dimer. Given the observational design, causality cannot be inferred; residual confounding may exist. The proposed risk prediction model requires external validation. Prospective randomized trials are needed to confirm these findings.

## Data Availability

The raw data supporting the conclusions of this article will be made available by the authors, without undue reservation.
