# Supplementary material for: Rivaroxaban plus antiplatelet therapy for coronary artery ectasia: 36-month outcomes and risk prediction from a retrospective cohort study
Source: Front Cardiovasc Med. 2026 Jul 15;13:1815929. doi: 10.3389/fcvm.2026.1815929 (PMC13416092; doi:10.3389/fcvm.2026.1815929)
Supplement: Supplementary file 1 [file Table1.docx]

**Supplementary Table S1. Descriptive Outcomes by Antiplatelet Agent (Aspirin vs. Clopidogrel) in the Study Group**

| **Outcome** | **Aspirin (n=94)** | **Clopidogrel (n=30)** | **P-value** |
| --- | --- | --- | --- |
| MACE at 36 months, n(%) | 8 (8.5) | 2 (6.7) | 0.99 |
| Major bleeding (BARC 3), n(%) | 1 (1.1) | 0 (0) | 0.99 |
| Any bleeding, n(%) | 21 (22.3) | 7 (23.3) | 0.99 |

**Supplementary Table S2. Landmark Analysis of MACE at 12, 24, and 36 Months**

| **Time point** | **Control group (n=124)** | **Study group (n=124)** | **HR (95% CI)** | **P value** |
| --- | --- | --- | --- | --- |
| 12 months | 12 (9.7%) | 4 (3.2%) | 0.33 (0.12–0.91) | 0.03 |
| 24 months | 18 (14.5%) | 7 (5.6%) | 0.36 (0.16–0.82) | 0.01 |
| 36 months | 27 (21.8%) | 10 (8.1%) | 0.34 (0.19–0.62) | <0.001 |

*Note: MACE, major adverse cardiovascular events. HR calculated using Cox proportional hazards model stratified by matched pairs. P value from log-rank test.*
